# *QuickStats:* Percentage of Children Aged 4–17 Years Who Had Ever Had Varicella (Chickenpox),* by Age Group — National Health Interview Survey, 2007–2016^†^

**DOI:** 10.15585/mmwr.mm6648a7

**Published:** 2017-12-08

**Figure:**
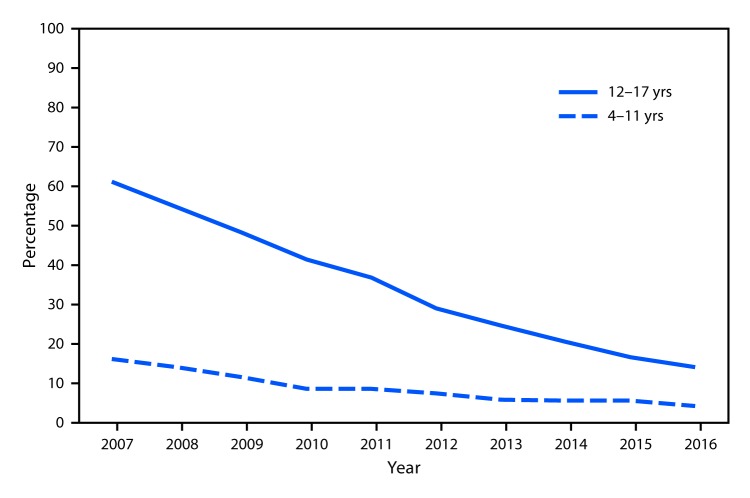
During 2007–2016, the percentage of children aged 4–17 years who had ever had chickenpox decreased among both younger children (aged 4–11 years) and older children (aged 12–17 years). Among younger children, the percentage of children who had ever had chickenpox declined by 73.9%, from 16.1% in 2007 to 4.2% in 2016. Among older children the percentage who had ever had chickenpox declined by 76.9%, from 61.4% in 2007 to 14.2% in 2016. During 2007–2016, older children were more likely than younger children to have ever had chickenpox.

**Source:** National Center for Health Statistics, National Health Interview Survey, 2007–2016. https://www.cdc.gov/nchs/nhis.htm.

